# Management of a Difficult-to-Treat Diabetic Foot Wound Complicated by Osteomyelitis: A Case Study

**DOI:** 10.1155/2020/3971581

**Published:** 2020-06-15

**Authors:** Maram Alkhatieb, Hatan Mortada, Hattan Aljaaly

**Affiliations:** ^1^Division of General Surgery, Department of Surgery, Faculty of Medicine, King Abdulaziz University, Jeddah, Saudi Arabia; ^2^Department of Plastic Surgery & Burn Unit, King Saud Medical City, Riyadh, Saudi Arabia; ^3^Division of Plastic Surgery, Department of Surgery, Faculty of Medicine, King Abdulaziz University, Jeddah, Saudi Arabia

## Abstract

**Introduction:**

Diabetic ulcers are a major health issue worldwide, causing significant economic burdens and affecting both the patient and the society as a whole. Predisposing factors in diabetic patients, known as the pathogenic triad, comprise trauma, ischemia, and neuropathy. Regardless of the cause, correct diagnosis and prompt treatment are essential in the management of leg ulcers. *Case History*. We report a case of a 51-year-old male patient, with a known history of type 2 diabetes mellitus who presented to our hospital with a history of two ulcers, one that he was mainly complaining of, which was actively infected and located at the posterior part of the distal left leg, and the second, dry ulcer caused by unrecognized trauma, located on the heel of the same limb. Magnetic resonance imaging showed osteomyelitis and degenerative changes in the calcaneonavicular and tarsal joints. The patient underwent multiple sessions of excisional debridement. He was started on negative pressure wound therapy with some improvements. However, after skin graft failure, Nanoflex powder was used, leading to complete wound closure within one month of treatment.

**Conclusion:**

A multidisciplinary holistic approach must be used when treating diabetic foot ulcers. Different modalities and sessions of debridement should be performed after optimizing the general condition of the patient.

## 1. Introduction

Wound healing is a complex process. It depends on multiple factors that play a major role in healing. The concentration of biochemical transmitters and the cellular composition of wound surfaces are the most important factors. A defect in one of these factors can lead to the development of skin ulcers, i.e., wounds that do not heal by the usual process. Undoubtedly, skin ulcers are caused by the alteration of physiological and functional integrates of the wounds. Regarding the types of ulcers, chronic ulcers are defined as wounds with more than 4-6 weeks of healing time [1]. One of the main contributing factors to wound healing is having risk factors, such as diabetes [2]. In addition, the most feared complication of diabetes mellitus is a diabetic ulcer [3]. Therefore, diabetic patients should maintain well-controlled blood glucose achieved by strictly adhering to medication therapy, exercise, and diet to decrease long-term complications, including ulcers [2]. Managing deep soft tissue wounds in diabetic patients who suffer from severe osteomyelitis can be difficult because of the complexity and magnitude of subsequent soft tissue defects [4–9]. In such cases, multidisciplinary and advanced wound care techniques, including negative pressure wound therapy (NPWT) and an ideal wound healing dressing are usually needed to enhance wound healing and increase the formation of granulation tissue [10–12]. In this case study, we report the use of Nanoflex powder (Altrazeal, Uluru, Inc., Addison, Texas, United States), which seems to fulfill the requirements of an ideal dressing to manage a difficult wound on the heel complicated by osteomyelitis, according to a previously published article which demonstrated lower leg surgical wound complete granulation and healing within 14 days of Nanoflex powder application [13]. Nanoflex is composed of 14.9% poly(2-hydroxypropyl methacrylate) (pHPMA), 84.8% poly(2-hydroxyethyl methacrylate) (pHEMA), and 0.3% sodium deoxycholate. When the powder is applied to a moist wound, the sterile powder interacts with the exudates within the wound, as its hydration causes the particles to combine and forms a flexible moist layer dressing. Nanoflex powder can be used in superficial acute wounds, surgical wounds, and chronic wounds, including pressure ulcers, leg ulcers, and diabetic foot ulcers [14].

## 2. Case Study

### 2.1. History

A 51-year-old Saudi male patient was presented to our outpatient Department at KAUH in March 2018, complaining of a 2-month history of ulcer. He had known uncontrolled type 2 diabetes mellitus for 26 years, dyslipidemia, and ischemic heart disease. He was obese and a known smoker for a couple of years. The ulcer was located at the distal left leg posteriorly. His wound might have started after trauma to his foot was sustained while climbing downstairs at work. He then noted a foul smell and discharge from a small ulcer that progressively got bigger during the last two months. The ulcer continued to discharge minimal amounts of fluid and pus despite different medical interventions, traditional herbal treatments, and oral antibiotics. He reported no fever, chills, sweating, malaise, rest pain, or intermittent claudication. He also denied weight loss, loss of appetite, night sweats, and other systemic manifestations.

### 2.2. Examination

**Figure 1:**
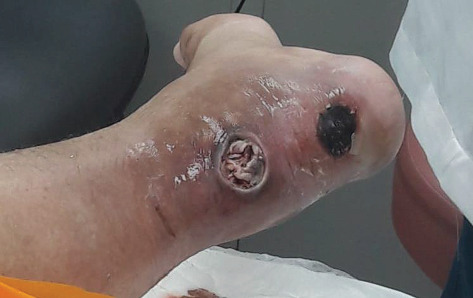
Wound presentation.

**Figure 2:**
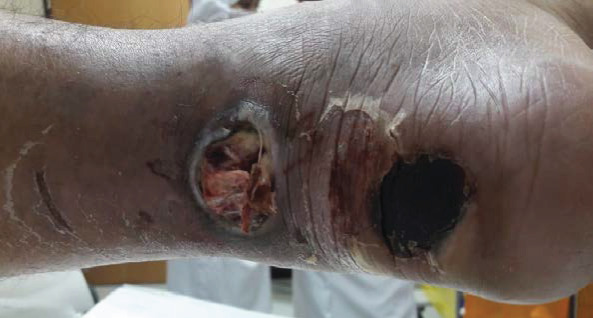
Wound presentation.

During the general examination, the patient was alert and oriented. He was morbidly obese (BMI 51.4 kg/m^2^). His vital signs were stable, and he was afebrile. On focus examination, the patient was found to have two ulcers; one ulcer was actively infected located at the distal left leg posteriorly around 5 cm from the heel and measured about 3 cm in diameter. It was circular with punched out edges, regular margins surrounded by erythematous skin with a necrotic floor with visible necrotic Achilles tendon. Visible signs of active local infection were noted, and it was the main reason for the clinic visit. The second ulcer was located posteriorly, on the heel of the left foot, 2 cm in diameter, black in color, was superficial and dry, circular with a regular margin, and with no signs of inflammation or infection (Figures 1 and 2).

Both lower limbs were dry and scaly (xerosis). Positive signs of trophic changes, brittle nails, and loss of hair were also observed. The vascular exam was fair. Capillary refilling was less than 2 seconds, and palpable distal pulses (dorsalis pedis and posterior tibial) were noted. The neurological exam showed bilateral neuropathic feet with the loss of protective sensation as demonstrated by proprioception and vibration testing using a 10 g monofilament (Semmes-Weinstein monofilament (SWM)). Furthermore, subluxation of the left ankle joint with a good passive range of motion was noted.

### 2.3. Provisional Clinical Diagnosis

The patient was provisionally diagnosed with localized deep tissue infection and abscess formation resulting in rupture of the Achilles tendon of the left lower limb.

### 2.4. Hospital Course

**Figure 3:**
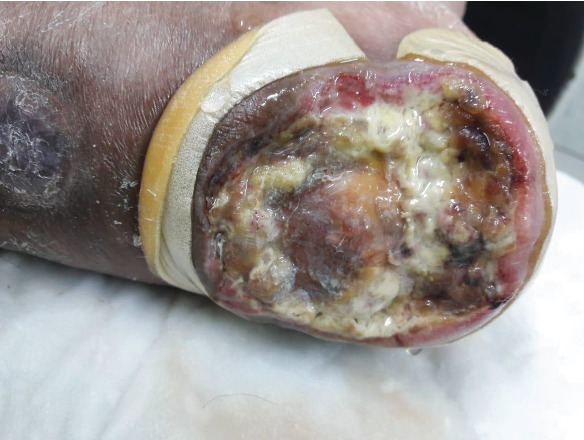
Distal wound progressively increasing in size.

**Figure 4:**
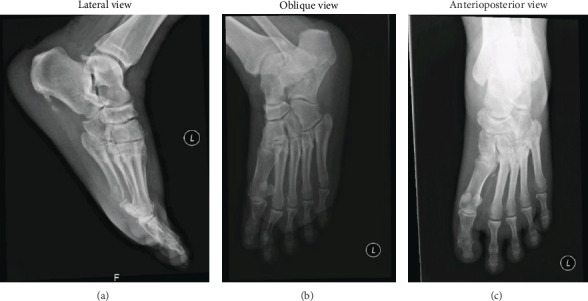


**Figure 5:**
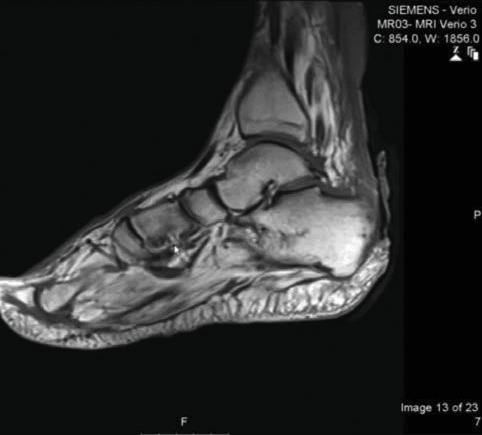
Lateral MRI view of left foot.

Once the patient was presented to the clinic, we started with local management, including debridement of sloughed soft tissue and irrigation; moreover, we initiated treatment with an empirical broad-spectrum antibiotic after wound cultures were obtained, in addition to all routine laboratory tests and X-ray images. His hemoglobin A1c was 11.38 mmol/L, urea (BUN) 6.5 mg/dL, Hb 10.9 g/dL, random glucose 19.2 mmol/L, C-reactive protein 28.7 mg/L, and WBC 11.62 × 10^9^/L. Initial foot and ankle X-ray was normal. He was referred to an endocrinologist to have better control of his blood sugar and to a plastic surgeon and orthopedic surgeon to have a multidisciplinary team approach. The patient's wound was dressed with a silver-containing dressing material to control his infection and wound discharge. The other wound was cleaned using normal saline and was dressed with iodine to keep the wound dry. He was followed up in the wound clinic. The dressing was changed every other day until culture results were released, and antibiotic was administered based on culture sensitivity. The first ulcer on the posterior left leg completely healed within 6 months. The other ulcer on the heel of the posterior left foot that progressively increased in size to up to 6 cm in diameter had part of the calcaneus exposed (Figure 3). Foot and ankle X-ray showed osteopenia, cortical loss, and periosteal reaction (Figures 4(a)–4(c)). Foot and ankle magnetic resonance imaging (MRI) showed an abnormal cortical enhancement of the posterior aspect of the calcaneus adjacent to the insertion of the Achilles tendon, overlying the wound, and associated with the posterior calcaneal cortical disruption. An enhancing rim indicating a collection was noted in the myotendinous junction of the Achilles tendon measuring approximately 4 × 2cm. Degenerative changes were noted in the calcaneonavicular and tarsal joints. These findings were observed with osteomyelitis of the calcaneus (Figure 5).

**Figure 6:**
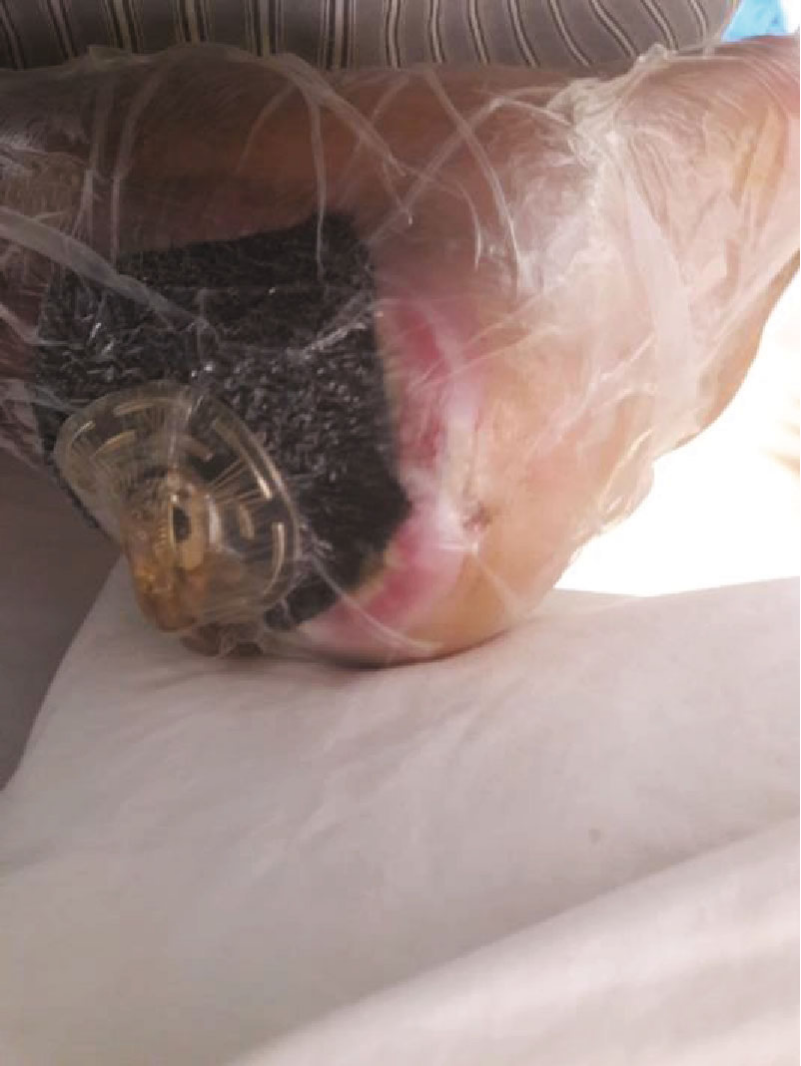
NPWT application.

**Figure 7:**
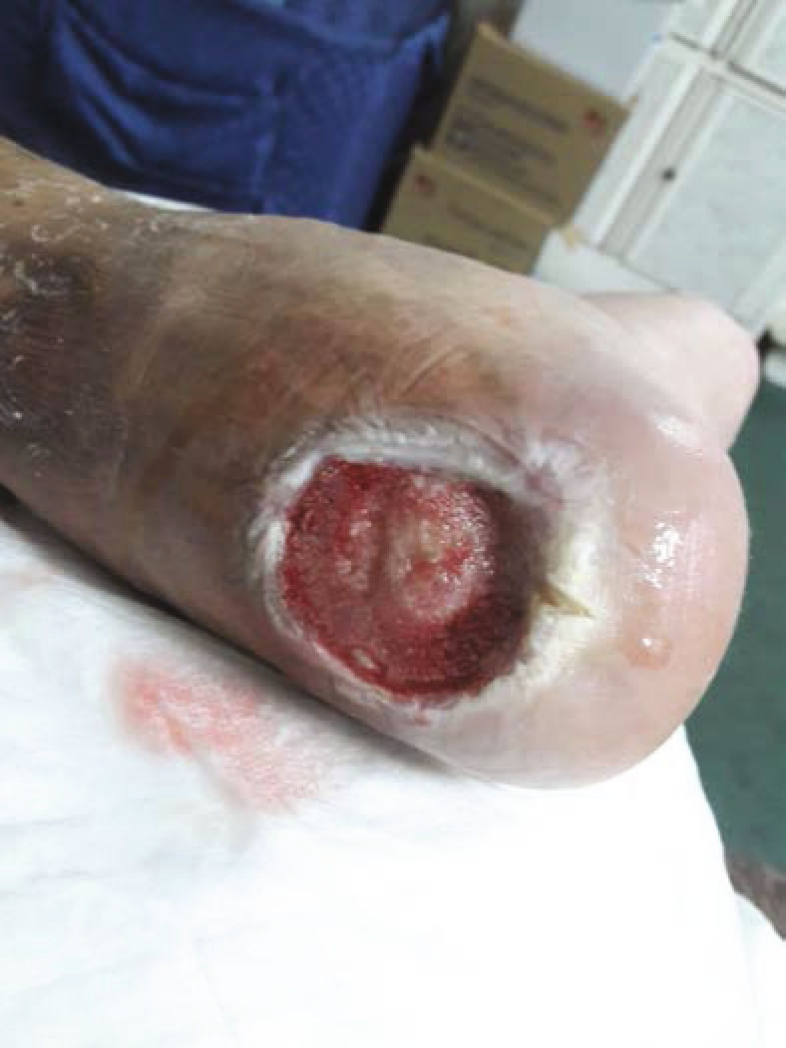
Granulation tissue developed after VAC.

The patient underwent multiple sessions of excisional debridement of the soft tissue and bone from the left foot. Moreover, we performed excisional debridement of the bone using a Volkmann bone curette. The patient was then started on negative pressure wound therapy (125 mmHg, continuous mode) for seven days. Improvements in wound healing were noticeable (Figures 6 and 7).

**Figure 8:**
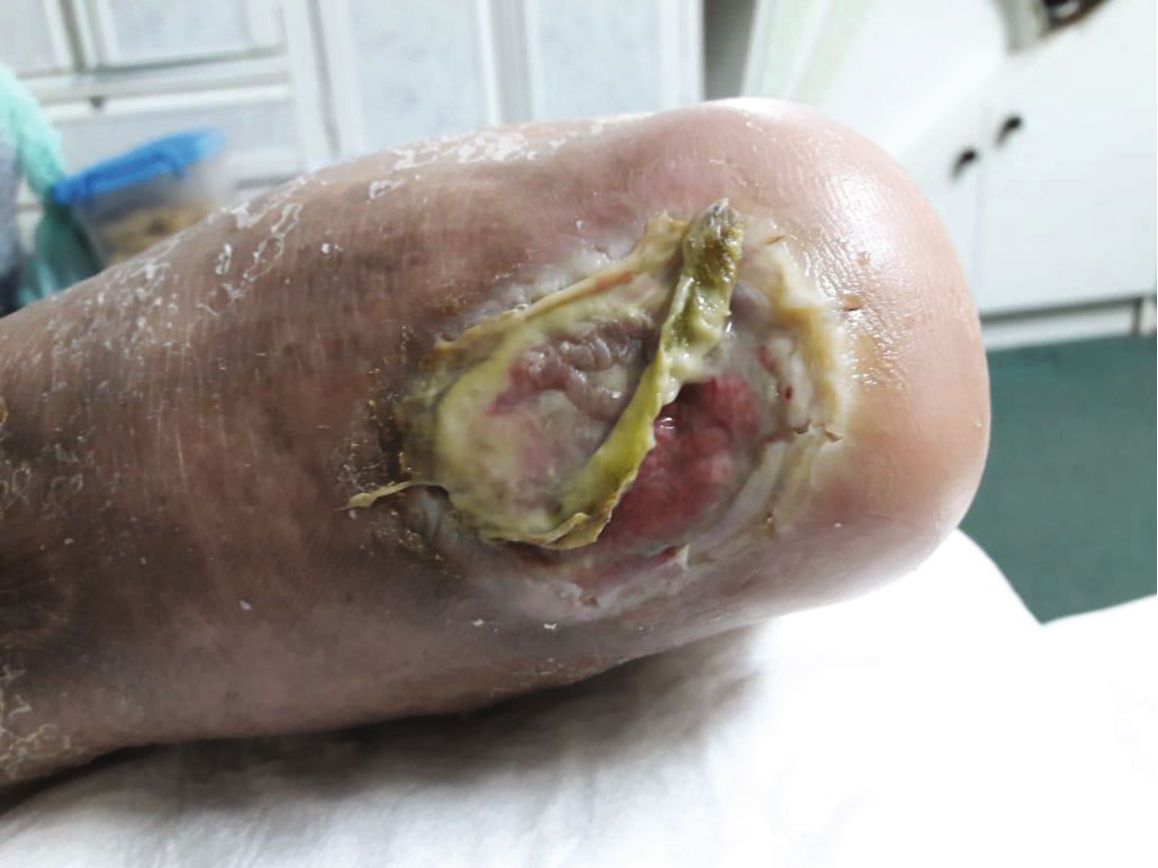
Failed skin graft after application.

The wound was seen by the plastic surgery team and planned for coverage using a split-thickness skin graft, which later failed after one week due to infection, although culture before the procedure was negative (Figure 8).

**Figure 9:**
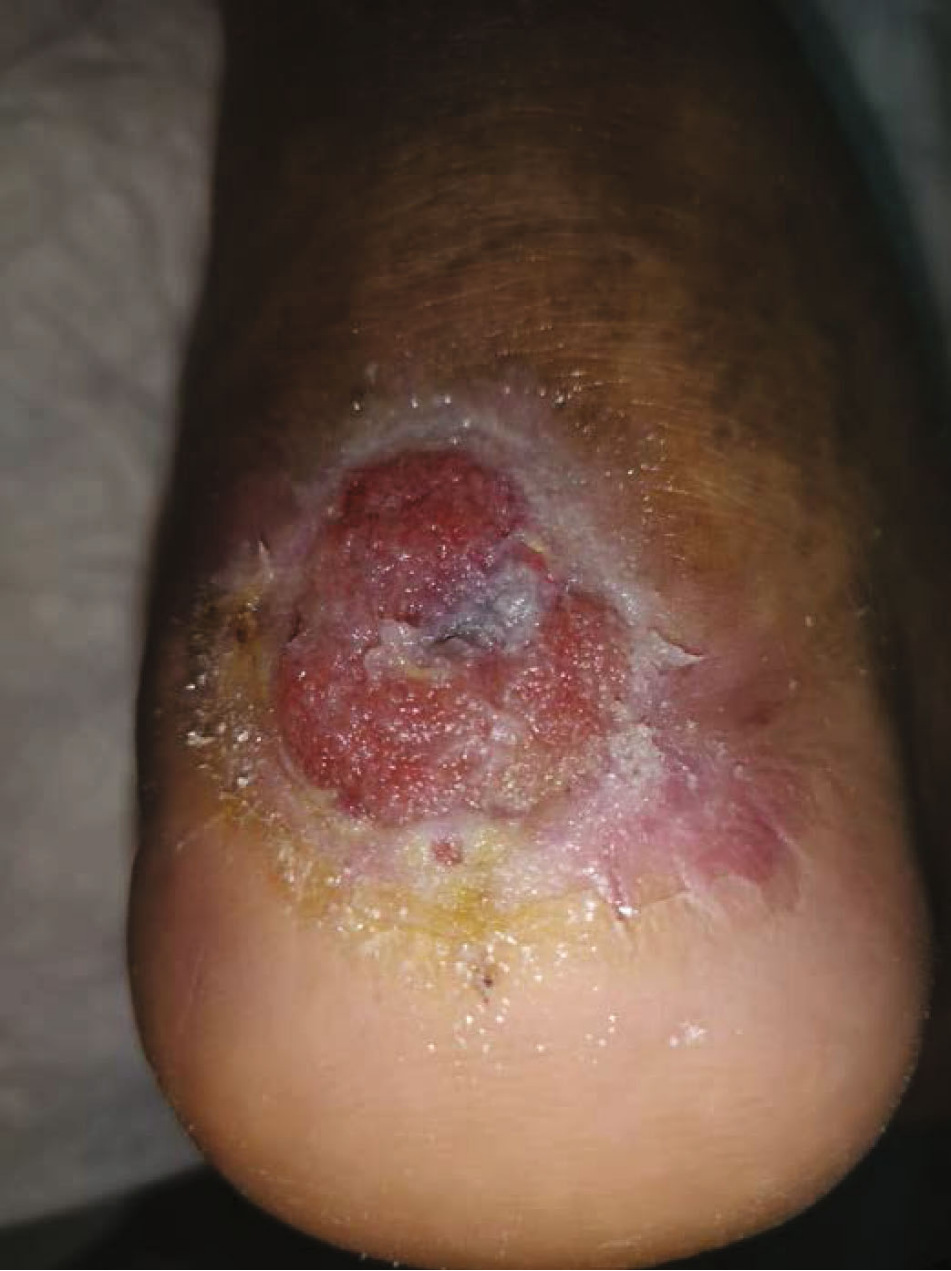
Nanoflex powder application.

**Figure 10:**
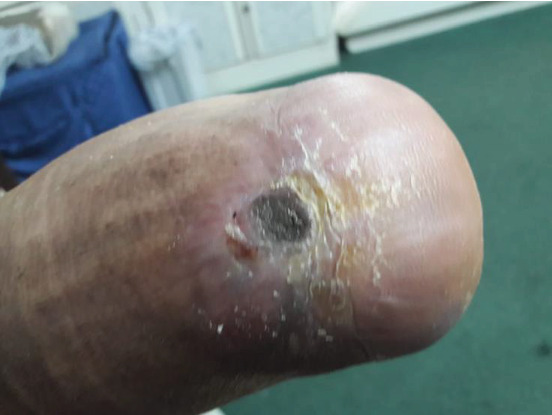
Wound completely closed after Nanoflex powder.

Lastly, Nanoflex powder (Altrazeal, Uluru, Inc., Addison, Texas, United States) was applied to the entire bed of the clean moist wound to form a thin, uniform layer; a spatula used to create an even matrix, and then saline was poured to accelerate dressing transformation (Figure 9). Finally, the wound was covered with a transparent film dressing, leading to complete wound healing within one month of treatment (Figure 10). Nanoflex powder was reapplied on the wound every 3-5 days for a month.

**Figure 11:**
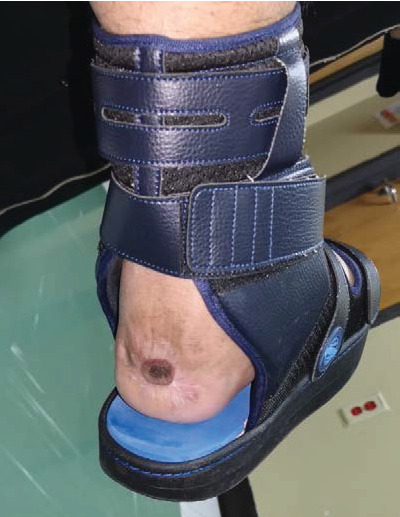
Rigid ankle brace was used to stabilize the ankle.

The patient was referred again to an orthopedic surgeon for fixing his ankle joint; however, he was not fit for Achilles tendon repair due to his general health condition, including morbid obesity, uncontrolled medical issues, and local causes such as a history of osteomyelitis. Therefore, a rigid ankle brace was used to stabilize his ankle (Figure 11).

## 3. Discussion

Diabetic ulcers are a major health issue worldwide, causing significant economic costs affecting both the patient and the society as a whole. The predisposing abnormalities in diabetic patients, known as the pathogenic triad, are composed of trauma, ischemia, and neuropathy. Diabetic foot occurs frequently because of neuropathic causes. Peripheral diabetic neuropathy is responsible for about 80% of amputations after a foot injury or a laceration [15]. Regardless of the cause, a correct diagnosis and prompt treatment are essential in the management of leg ulcers. Therefore, the management of diabetic foot and ulcer should involve a mix of strategies, including patient care and education. Moreover, adherence to physician recommendations and routine inspections of foot, skin, and toenails is strongly encouraged. In addition, tight glycemic control by oral hypoglycemic drugs or insulin is needed [16–18]. One of the most important parts of diabetes management is the holistic, integrated, and multidisciplinary nature of diabetes care, which provides diabetes support, and education, in addition to care. When it comes to dealing with chronic leg ulcers, patients consult different specialists, including endocrinologist for proper glycemic control, diabetic foot surgeon and plastic surgeon for wound coverage, vascular surgeon for vascular complications and, lastly, infectious disease specialist for optimum antibiotic treatment. Chronic leg ulcers are common complications of diabetes, usually associated with a significant impact on the quality of life. These chronic leg ulcers can be a burden to the healthcare systems [19–21]. Similarly, they also can be associated with devascularization, infections, neuropathies, and prolonged decubitus. Like in our case, several factors can contribute to the etiology of chronic leg ulcers and can significantly delay wound healing [22]. In our case, the patient underwent several sessions of debridement for osteomyelitis. Also, a split-thickness skin graft was done, although it failed within the first week of application. After that, NPWT was reapplied for five days, and then lastly, we transitioned to Nanoflex crystals dressing as our last solution in dealing with the patient. However, we cannot always go with skin grafts as initial management when dealing with a patient with multiple comorbidities.

When the powder is applied to the moist wound bed, the sterile material powder interacts with an ionized fluid such as exudate, saline, or blood. The powder then converts to an aggregated exudate-controlling wound dressing. After aggregation, the Nanoflex wound dressing conforms to the topography of the wound bed, fills the dead spaces, and seals the wound margins. In our case, the patient continued using the powder dressing after discharge. The wound was checked continuously every week at the time of dressing change, and Nanoflex powder was applied every 3-5 days for nearly 1 month. Interestingly, the dead space kept decreasing in size during consecutive visits, with infiltrating granulation tissue. Complete wound healing was seen within a month. However, the patient had 10 months of treatment, starting from first presentation until the last follow-up. He was admitted to the hospital thrice, and each time, for less than 2 weeks. So, the total hospital stay was about 36 days. Our case highlights a complicated ulcer on the heel that started secondary to a simple trauma and went unnoticed because of underlying neuropathy caused, in part, by associated comorbidities, long-term medication noncompliance, and poor glycemic control. This case would have likely led to leg amputation; however, due to the collaborative effort of the management team who used different modalities of care and wound management, amputation could be avoided after treatment of the patient's osteomyelitis.

## 4. Conclusion

We present this case to highlight the positive outcomes that could result when a multimodal approach is used to treat a complicated wound. A multidisciplinary holistic team approach is required to treat a diabetic ulcer. Besides, the use of different modalities and debridement sessions should be done after optimizing the general medical condition of the patient, including nutritional status and reasonably good glycemic control.
